# GSDMD contributes to myocardial reperfusion injury by regulating pyroptosis

**DOI:** 10.3389/fimmu.2022.893914

**Published:** 2022-09-23

**Authors:** Xiaomiao Ye, Peng Zhang, Yuting Zhang, Jingyun Luan, Caili Xu, Zhengyu Wu, Dianwen Ju, Wei Hu

**Affiliations:** ^1^ Department of Cardiology, Minhang Hospital, Fudan University, Shanghai, China; ^2^ Department of Biological Medicines & Shanghai Engineering Research Center of Immunotherapeutics, Fudan University School of Pharmacy, Shanghai, China; ^3^ TAU Cambridge Ltd, Cambridge, United Kingdom; ^4^ Minhang Hospital, Fudan University, Shanghai, China

**Keywords:** gasdermin D, myocardial reperfusion injury, pyroptosis, interleukin 1β, interleukin 18

## Abstract

**Background:**

Gasdermin D (GSDMD) plays an essential role in the pathway of pyroptosis. However, whether GSDMD participates in myocardial ischaemia/reperfusion injury (MI/RI) remains poorly understood.

**Methods:**

Serum levels of GSDMD and IL-18 in ST-segment elevation myocardial infarction (STEMI) patients were measured by ELISA. The expression of GSDMD and GSDMD N-terminal (GSDMD-NT) *in vivo* and *in vitro* was assessed by western blot and immunofluorescence staining. GSDMD^-/-^ mice and wild type (WT) mice were induced MI/RI, followed by cardiac ultrasound and histological analysis.

**Results:**

Clinically, patients suffering from STEMI after percutaneous coronary intervention (PCI) exhibited higher levels of GSDMD and IL-18 than that in the controls. *In vitro*, the cleavage of GSDMD was significantly upregulated in macrophages exposed to hypoxia/reoxygenation or H_2_O_2_. *In vivo*, the levels of GSDMD and GSDMD-NT increased notably after MI/RI, especially in macrophages infiltrating in the infarct area. Moreover, compared with WT mice, GSDMD^-/-^ mice showed reduced infarct size (25.45 ± 3.07% versus 36.47 ± 3.72%), improved left ventricular ejection fraction (37.71 ± 1.81% versus 29.44 ± 2.28%) and left ventricular fractional shortening (18.01 ± 0.97% versus 13.62 ± 1.15%) as well as attenuated pathological damage after I/R injury, along with reduced levels of proinflammatory cytokines and decreased infiltration of neutrophils.

**Conclusions:**

Our study revealed that GSDMD deficiency significantly alleviated the inflammatory response by regulating pyroptosis, reduced the infarct size and preserved cardiac function after MI/RI, thus providing a potential strategy for the treatment of myocardial reperfusion injury.

## Introduction

Acute myocardial infarction (AMI) resulted from sudden coronary artery occlusion causes high level of mortality worldwide (1, 2). Restoring blood flow as soon as possible is the most important and urgent treatment to limit the infarct area and improve cardiac function. However, sudden reperfusion of the ischaemic area exacerbates myocardial injury and further increases the infarct size by half, which is known as myocardial ischaemia/reperfusion injury (MI/RI) (2, 3). Multiple studies have demonstrated that the inflammatory response, calcium overload and oxidative stress are involved in this complex and dynamic process and induce various modes of cardiomyocyte death (2, 4, 5). However, due to various reasons, such as the significant differences between animal models and AMI patients, many effective therapeutic strategies in the laboratory have been proved limited in clinical efficacy (5–10). Therefore, the underlying mechanisms and new effective therapeutic strategies are urgent to be explored.

Inflammatory response plays a pivotal role in the MI/RI (11). Damage to the myocardium triggers a robust inflammatory cascade with immune cell infiltration and proinflammatory cytokine release. Pyroptosis, a proinflammatory form of programmed cell death, is characterized by activated caspase-1 and the release of mature IL-1β and IL-18 (12). The injured myocardium releases various damage-associated molecular patterns (DAMPs), which are detected by sensor molecules on the cell surface and prompt the transcription of NLRP3 and pro-IL-1β (13, 14). Secondary signals, such as ATP or reactive oxygen species (ROS), activate inflammasomes and initiate the self-cleavage of caspase-1 and the maturation of pro-IL-1β and pro-IL-18 (15). However, these mature cytokines are not released through the classic endoplasmic reticulum-Golgi secretion pathway (16). According to recent studies (17, 18), cytosolic GSDMD may be a key effector protein in the formation of specific channels for the release of these cytokines.

GSDMD, the executor of pyroptosis, is mainly expressed in immune cells and intestinal epithelial cells (19), and can be cleaved by activated caspase-1 or other inflammatory caspases to generate N-terminal p30-GSDMD (GSDMD-NT), which subsequently oligomerizes, translocates and perforates the cell membrane (20). The oligomerization of GSDMD-NT is inhibited by its C-terminal portion (21). Due to the inner diameter of the pores, IL-1β and IL-18 can be released into the extracellular matrix (22, 23). The pathological role of GSDMD has been validated in many inflammatory diseases, such as inflammatory bowel disease (24, 25), autoimmune encephalitis (26), and alcoholic steatohepatitis (27), as well as various I/R injury models of the brain (28), liver (29, 30) and intestine (31). The researches showed that GSDMD played an role in AMI (32) and MI/RI (33, 34). However, the underlying specific mechanisms of GSDMD in MI/RI still remains unclear.

In this study, we investigated the expression of GSDMD and characterized its role in MI/RI employing GSDMD deficient mice. We demonstrated that the loss of GSDMD ameliorated MI/RI by reducing the release of inflammatory cytokines and the infiltration of neutrophils. We proposed that GSDMD may provide a new target for the treatment of MI/RI.

## Materials and methods

### Materials

Antibodies against GSDMD (cat: ab209845), caspase-1 (cat: ab179515) and F4/80 (cat: ab100790) were from Abcam (Cambridge, UK), antibodies against β-actin (cat: GB12001) and Ly-6G (cat: GB11229) were from Servicebio (Wuhan, China), and antibodies against NLRP3 (cat: 15101) and IL-1β (cat: 12242) were from Cell Signaling Technology (Danvers, USA). The IL-1β, IL-18 and IL-6 of mouse ELISA kits and IL-18 of human ELISA Kit were from MultiSciences (Hangzhou, China), and the GSDMD of human ELISA kit (ab272463) was from Abcam. 2,3,5-triphenyltetrazolium chloride (TTC, cat: T8877) and Evans blue (cat: T2129) were from Sigma-Aldrich (St Louis, USA). DCFH-DA (cat: HY-D0940) and disulfiram (cat: HY-B0240) were from MedChemExpress (State of New Jersey, USA). N-acetyl-L-cysteine (cat: S0077) was from Beyotime Biotechnology (Shanghai, China).

### The recruitment of control patients and STEMI patients

Serum samples were obtained from control patients and 29 STEMI patients before and after PCI. The STEMI patients were diagnosed by clinical manifestations, laboratory examinations, electrocardiography and coronary angiography. The average time from symptom onset to reperfusion was 5.56 ± 1.16 hours and the blood flow on reperfusion was evaluated as TIMI 3. Patients who felt chest discomfort but had normal coronary arteries assessed by coronary angiography were included in the control group. Our research was approved by the Institutional Research Ethics Committee of Shanghai Minhang Hospital (Shanghai, China), and written informed consent from each patient was obtained.

### Cell culture and treatments

Ana-1 cells, J774a.1 cells and H9c2 cells were obtained from the Chinese Academy of Sciences (Shanghai, China) and cultured according to standard protocols. The adult primary cardiomyocytes were isolated and cultured according to the research (35). To induce I/R *in vitro*, cells were placed in glucose- and serum-free DMEM and incubated in a hypoxic culture chamber (1% O_2_, 5% CO_2_ balanced with N_2_) for 6 hours. Then, the medium was replaced with high-glucose DMEM, and the cells were moved to a normal incubator (95% air and 5% CO_2_) for at least one more hour. The cells were photographed by the inverted immunofluorescence microscope (Olympus Corporation, Japan). In addition, Ana-1 cells were challenged with normal DMEM containing 100 μM or 200 μM H_2_O_2_ for six hours to mimic oxidative stress. Besides, DMEM containing 5mM NAC or 5μM disulfiram were used to culture cells for 2 hours before H_2_O_2_ stimulation. The level of LDH release in the supernatant was measured by CytoTox 96^®^ Non-Radio Cytotoxicity Assay (cat: G1781, Promega, Wisconsin, USA) to evaluate the cell death. The level of cellular ROS was stained with DCFH-DA and measured by flow cytometry (36).

### Western Blot

Protein was isolated from heart tissues and cultured cells, processed as previously described (37), and incubated with specific antibodies against GSDMD, NLRP3, mature IL-1β, ASC and cleaved caspase-1. Afterwards, ECL reagent (Meilunbio, Dalian, China) was used to visualize the specific binding, and semiquantitative analysis was performed by ImageJ software (National Institutes of Health, Bethesda, MD, USA).

### Mice

Mice were housed under specific pathogen-free (SPF) conditions, and all animal experiments were carried out with the approval of the Animal Ethics Committee of the School of Pharmacy at Fudan University. Wild-type (WT) C57BL/6 mice (22-27 g, male, 10–14 weeks old, SPF Biotechnology Company, Beijing, China) served as controls. GSDMD deficient mice were generated as previously described (23).

### The myocardial I/R model

First, the mice were anaesthetized with 1.5% isoflurane gas. Then, we disinfected the left side of the chest, made a vertical incision, opened the third intercostal muscle and pushed the heart out. The left anterior descending coronary artery (LAD) was exposed and immediately ligated. Visual observation of myocardial blanching was an indication of successful modelling. After 45 minutes of myocardial ischaemia, the slip knot was loosened. The mice in sham group underwent the same surgical procedures without tying the slipknot. The mice were sacrificed at specific time points, and then serum and heart tissue were collected for further analysis.

### Echocardiography

Echocardiography was performed on the third day after surgery. Mice were anaesthetized by inhaling isoflurane, and the heart rate was maintained above 400 bpm. M-mode images were acquired with a 40 MHz probe on a Vevo 2100 (Visual Sonics Inc., Toronto, Canada). The left ventricle ejection fraction (LVEF) and left ventricle fractional shortening (LVFS) were determined on the instrument in a blinded manner.

### Evans blue and TTC staining

On the first day after surgery, the LAD was occluded again, and 2% Evans blue was injected into the left ventricle (LV) until the heart turned blue. The heart was frozen immediately in dry ice and excised into five-to-six slices, which were incubated in 1.5% TTC solution at 37 °C for 15-20 minutes, fixed in 5% paraformaldehyde overnight, placed on paper and finally photographed by a digital camera. The area at risk (AAR) and infarct area (IA) were analyzed by ImageJ software.

### Immunohistochemistry (IHC) and immunofluorescence (IF) analysis

Before further immunostaining, heart tissue was assessed by haematoxylin and eosin (H&E) staining. Deparaffinization and antigen retrieval were performed on paraffin-embedded tissue sections to prepare for subsequent staining. IHC staining of Ly-6G (1:200) with haematoxylin was used to assess the infiltration of neutrophils. For IF analysis, the heart sections were incubated with anti-F4/80 and anti-GSDMD primary antibodies, counterstained with Hoechst 33342 and imaged by the LSM710 confocal microscope (ZEISS, Jena, Germany).

### ELISA

The levels of target cytokines in mouse or patient serum were evaluated by commercial ELISA kits according to the instructions.

### mRNA sequencing analysis

Total RNA was extracted from the infarct area on the third day after MI/RI. RNA concentration, integrity and purity were assessed. Then, mRNA was separated, broken into short fragments and synthesized into double-stranded cDNA, which was purified using AmpureBeads (Beckman, California, America). Then, dA-tails and sequencing connectors were added to the end-repair-product of the cDNA. Purification and screening were performed by AmpureBeads. PCR was performed again to obtain the cDNA library. Finally, mRNA sequencing was performed on a Nova 6000 Sequencer (Illumina, California, America).

### Statistical analysis

All quantitative results were presented as the mean ± SEM or median with interquartile ranges. Student’s *t* tests or the Mann-Whitney U test were employed to perform comparisons between two groups. These analyses were realized by SPSS version 20. *P < 0.05 and **P < 0.01 indicated the statistical significance.

## Results

### The level of GSDMD further increased in STEMI patients after PCI

**Figure 1:**
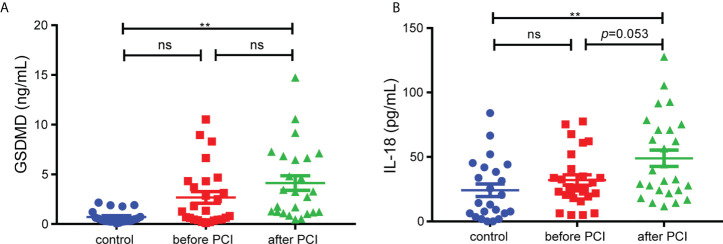
The serum levels of GSDMD. **(A)** and IL-18 **(B)** in STEMI patients (n=29) before and after PCI and control subjects (n=23). Comparisons were performed by one-way ANOVA with *post-hoc* tests (** and ns indicated *p*<0.01 and not significant, respectively).

Our study included 29 STEMI patients and 23 control subjects and the baseline characteristics of the patients were shown in **Supplemental Table 1**. We collected the serum from patients to figure out the levels of GSDMD and IL-18 across the entire process of ischemia and reperfusion. The serum level of GSDMD was not higher in STEMI patients before PCI than in controls. Although there was only minor difference between before PCI and after PCI, the level of GSDMD was higher in STEMI patients after PCI than in controls (*p* < 0.01) (**Figure 1A**). Meanwhile, the serum level of IL-18, a key player in pyroptosis, was also measured. Similar to the changes in GSDMD, serum IL-18 in STEMI patients after PCI was increased compared with controls (**Figure 1B**). These results suggested that pyroptosis and GSDMD might be involved in whole process of MI/RI.

### H/R induced macrophages pyroptosis *in vitro* with the cleavage of GSDMD

**Figure 2:**
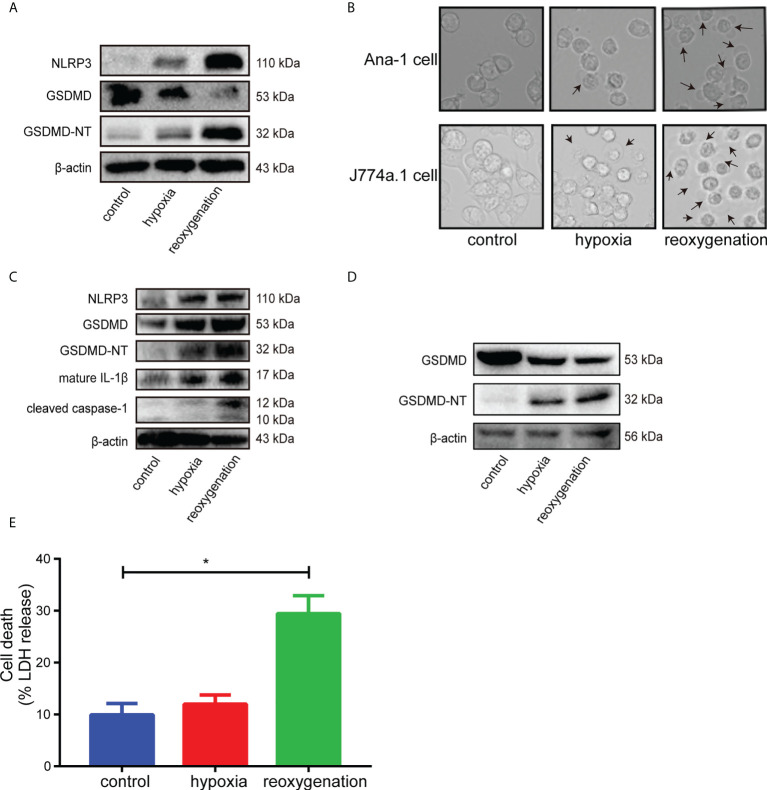
**(A)**, Immunoblot analysis of NLRP3, GSDMD and GSDMD-NT protein expression in adult primary cardiomyocytes subjected to hypoxia for 6 hours and reoxygenation for 12 hours. **(B)**, Representative images of Ana-1 cells and J774a.1 cells after different treatments were acquired by microscopy. **(C)**, Representative Western blots showing NLRP3, GSDMD, GSDMD-NT, mature IL-1β and cleaved caspase-1 in Ana-1 cells treated with hypoxia for 6 hours and reoxygenation for 1 hour. **(D)**, Representative Western blots showing GSDMD, GSDMD-NT in J774a.1 cells treated with hypoxia and reoxygenation. **(E)**, The level of LDH released in the supernatant from Ana-1 cells after H/R. (* indicated *p*<0.05).

First, H9c2 cardiomyocytes were stimulated with hypoxia/reoxygenation (H/R), and the results of western blot showed that the levels of GSDMD-NT, NLRP3 and mature IL-1β increased at different time points after reoxygenation (**Figure S1**). H9c2 cells, the modified cell line derived from embryonic rat heart tissue, shared some properties with skeletal muscle and could transdifferentiate into skeletal myotubes in no-serum medium (35, 38). The metabolism and voltage-dependent calcium channels of H9c2 cells varied when in different differentiation stage, which would influence the real results of interventions. Besides, the gene expression in H9c2 cells was extremely different from adult cardiomyocytes, which would cause the unsuccessful clinical translation of cardioprotection interventions because AMI was almost exclusively in the adult population (38, 39). Therefore, due to the substantial limitations of H9c2 cells for cardioprotection research, the freshly isolated adult cardiomyocytes, the ideal gold standard for H/R experiments, was adopted to stimulated with H/R. Western blot analysis showed that the levels of GSDMD-NT and NLRP3 increased after reoxygenation in adult primary cardiomyocytes (**Figure 2A**). GSDMD is mainly expressed in macrophages and intestinal epithelial cells (19). Ana-1 cells and J774a.1 cells became round and showed blebbing after reoxygenation compared with the control group (**Figure 2B**). The expression levels of NLRP3, GSDMD-NT, cleaved caspase-1 and mature IL-1β rose significantly in Ana-1 cells exposed to H/R (**Figure 2C**). Meanwhile, the level of GSDMD-NT was upregulated in J774a.1 cells after reoxygenation (**Figure 2D**). Furthermore, the level of LDH in the supernatant was markedly increased in Ana-1 cells after H/R (**Figure 2E**). These results showed that H/R could induce pyroptosis in macrophages with GSDMD cleavage.

### Oxidative stress induced macrophages pyroptosis *in vitro*

**Figure 3:**
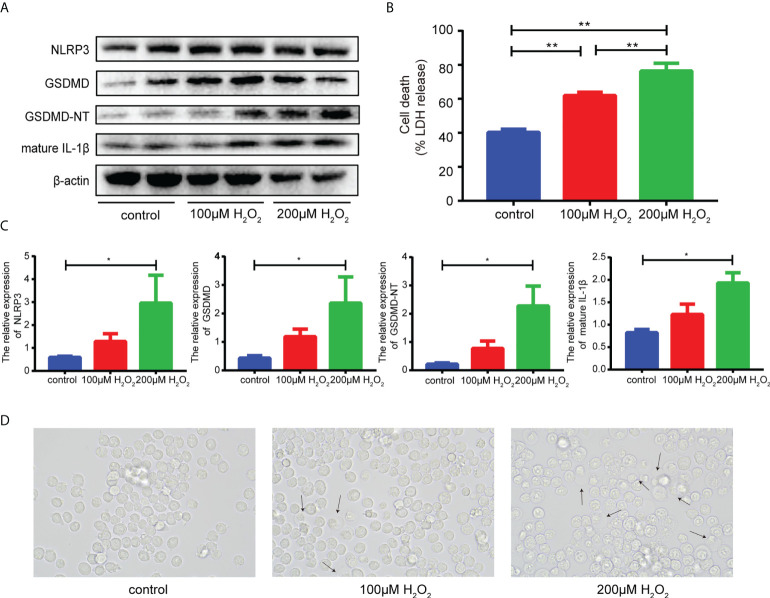
**(A, C)**, Representative Western blots **(A)** and averaged data **(C)** of NLRP3, GSDMD, GSDMD-NT and mature IL-1β in Ana-1 cells treated with H_2_O_2_. **(B)**, The level of LDH released in the supernatant from Ana-1 cells exposed to H_2_O_2_. **(D)**, Representative images of Ana-1 cells after H_2_O_2_ treatment. (* and ** indicated *p*<0.05 and *p*<0.01, respectively).

H_2_O_2_ was used to simulate oxidative stress during MI/RI (40, 41). Compared with the control group, Ana-1 cells stimulated with H_2_O_2_ exhibited increased levels of GSDMD-NT, mature IL-1β and NLRP3 compared with the control group. (**Figures 3A, C**), higher levels of LDH release (**Figure 3B**) and significant morphological changes (**Figure 3D**). Moreover, compared with the effects of low-dose H_2_O_2_, these changes were more obvious in response to high-dose H_2_O_2_. However, the relationship between oxidative stress and pyroptosis in MI/RI remained unclear. So N-acetyl-L-cysteine (NAC), an oxidative stress inhibitor, and disulfiram (DSF) inhibiting GSDMD-mediated pyroptosis and inflammatory cytokines release (42)were used to figure it out. The western blot showed that the levels of NLRP3, GSDMD-NT and cleaved caspase-1 reduced when NAC was added after H_2_O_2_ stimulation, which indicated that inhibiting oxidative stress would reduce pyroptosis (**Figures S2A, S2B**). The results of flow cytometry showed that the level of cellular ROS increased after H_2_O_2_ stimulation and reduced when added with disulfiram, suggesting that inhibiting pyroptosis would alleviate oxidative stress (**Figures S2C, S2D**). Therefore, these results suggested oxidative stress and pyroptosis were relatively independent but affected each other in MI/RI.

### The expression level of GSDMD increased after MI/RI

**Figure 4:**
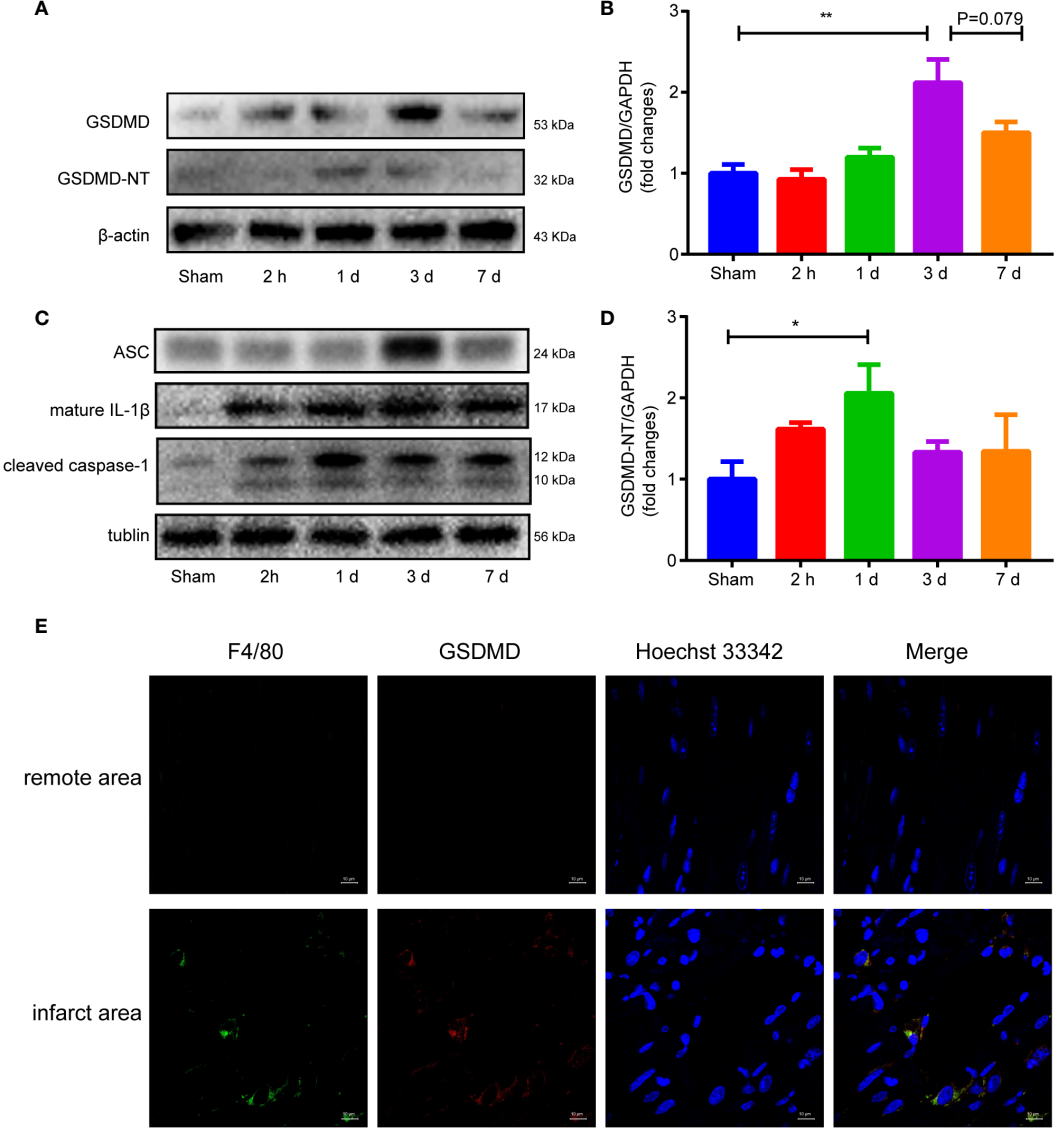
**(A, B, D)**, Immunoblot analysis **(A)** and averaged data **(B, D)** of GSDMD and GSDMD-NT in the infarct area of heart tissue at different time points after myocardial reperfusion injury. **(C)** Representative Western blots showing ASC, mature IL-1β and cleaved caspase-1 in the infarct area of heart tissue at different time points after MI/RI. **(E)** Immunofluorescence staining of GSDMD and F4/80 in heart tissue on Day 1 after I/R. (* and ** indicated *p*<0.05 and *p*<0.01, respectively).

Western blot analysis showed that the GSDMD-NT increased as early as 2 hours after reperfusion, peaked on Day 1, and then gradually decreased until Day 7 when it approached baseline levels (**Figures 4A, D**). The expression of GSDMD showed a different pattern and peaked on the third day after surgery (**Figures 4A, B**). The other proteins in the pyroptosis pathway, such as ASC, cleaved caspase-1 and mature IL-1β, all increased after surgery (**Figure 4C**). Immunofluorescence staining demonstrated obvious colocalization of GSDMD and F4/80, suggesting that GSDMD was mainly expressed in F4/80^+^ macrophages infiltrating in the infarct area (**Figure 4E**), which was consistent with previous studies (19, 31).

### GSDMD deficiency attenuated MI/RI

**Figure 5:**
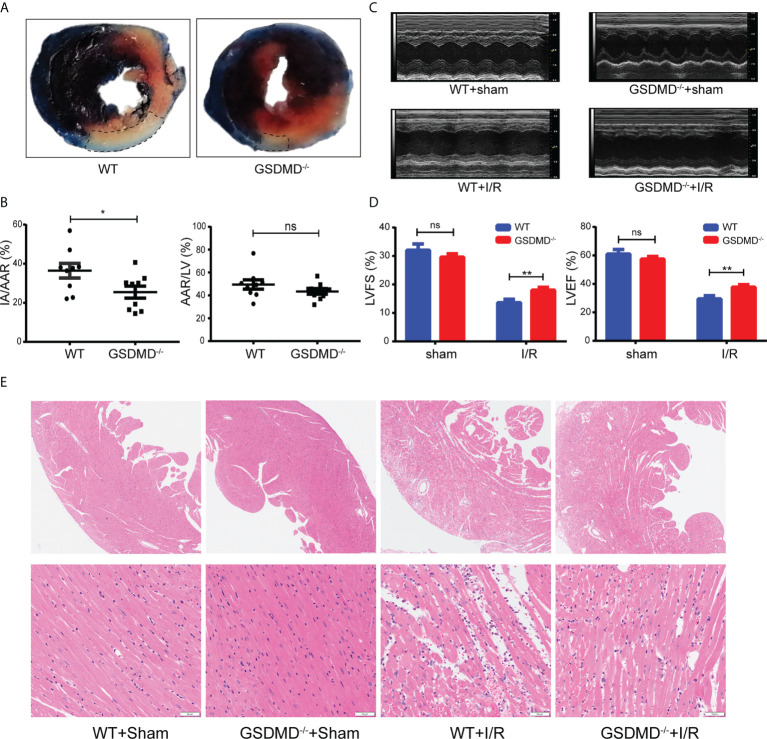
**(A)**, Representative images showing heart tissues from both WT mice and GSDMD-/- mice after Evans blue-TTC staining on Day 1 after I/R. **(B)**, The average AAR/LV and IA/AAR in WT mice and GSDMD^-/-^ mice (n=8-10). **(C, D)**, M-mode echocardiographic images **(C)** and analysis of LVEF and LVFS **(D)** in WT mice and GSDMD^-/-^ mice on Day 3 after I/R injury or sham surgery. **(E)** Representative H&E staining of heart tissue from WT mice and GSDMD^-/-^ mice on Day 1 after I/R injury or sham operation. (ns, * and ** indicated not significant, *p*<0.05 and *p*<0.01, respectively).

To illustrate the effect of GSDMD on MI/RI, GSDMD^-/-^ mice and WT mice were used to establish myocardial reperfusion injury, and then infarct size, heart function and pathological damage were assessed. Because the highest level of GSDMD-NT occurred on the first day after I/R, we performed Evans blue/TTC double staining at that time to assess the extent of injury. With a similar AAR/LV, the IA/AAR was much lower in GSDMD^-/-^ mice than in WT mice (36.47 ± 3.724% versus 25.45 ± 3.07%) (**Figures 5A, B**). On the third day after reperfusion, echocardiography was performed to measure LVEF and LVFS and evaluate the effect of GSDMD on cardiac function. Compared with those in the sham group, mice that underwent I/R exhibited extremely decreased LVEF and LVFS. As shown in **Figures 5C, D**, the LVEF and LVFS were significantly higher (37.71 ± 1.81% versus 29.44 ± 2.28%, 18.01 ± 0.97% versus 13.62 ± 1.15%, respectively) in GSDMD^-/-^ mice after I/R than in WT mice, suggesting that the loss of GSDMD could alleviate the impairment in cardiac function after I/R. This finding was further confirmed by histopathological alterations, as shown by H&E staining. Compared with sham-operated WT mice, post-I/R WT mice had disorganized cardiomyocytes and robust infiltration of inflammatory cells and erythrocytes, but these effects were significantly ameliorated in the GSDMD deficient group, which indicated the alleviation of myocardial injury (**Figure 5E**).

### GSDMD deficiency alleviated the inflammatory response

**Figure 6:**
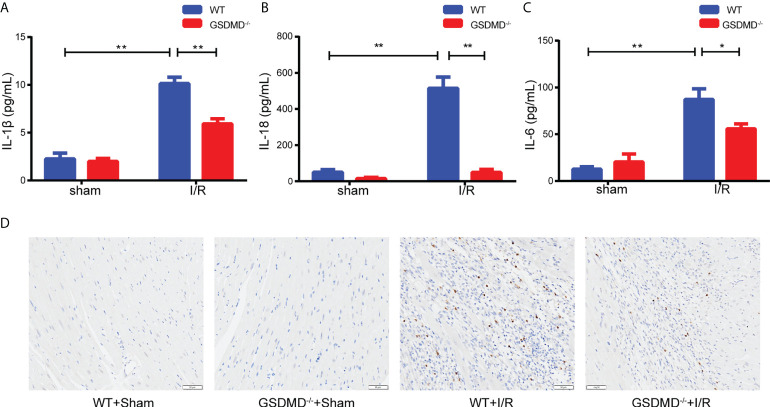
**(A–C)**, Serum levels of IL-1β **(A)**, IL-18 **(B)** and IL-6 **(C)** in WT mice and GSDMD^-/-^ mice on Day 3 after I/R injury or sham surgery. **(D)** Representative images showing immunohistochemical staining of heart tissue from WT mice and GSDMD^-/-^ mice on Day 3 after I/R injury or sham operation. (* and ** indicated *p*<0.05 and *p*<0.01, respectively).

Compared with those in the sham group, the serum levels of IL-1β (**Figure 6A**) and IL-18 (**Figure 6B**) in WT mice increased significantly after I/R, but were notably reduced in the GSDMD^-/-^ group. In addition, we measured the level of IL-6, a critical proinflammatory cytokine, which was markedly higher in WT mice than in GSDMD^-/-^ mice after I/R (**Figure 6C**). Furthermore, the IHC results showed that the infiltration of Ly-6G^+^ neutrophils was much more in WT mice after I/R than in sham mice, but this effect was strikingly suppressed in GSDMD^-/-^ mice (**Figure 6D**), suggesting that GSDMD deficiency inhibited the infiltration of neutrophils. Our results showed that the inflammatory response was alleviated in GSDMD^-/-^ mice after I/R compared to WT mice, which was consistent with the results of echocardiography and Evans Blue/TTC double staining.

### GSDMD deficiency regulated the inflammatory response and heart function

**Figure 7:**
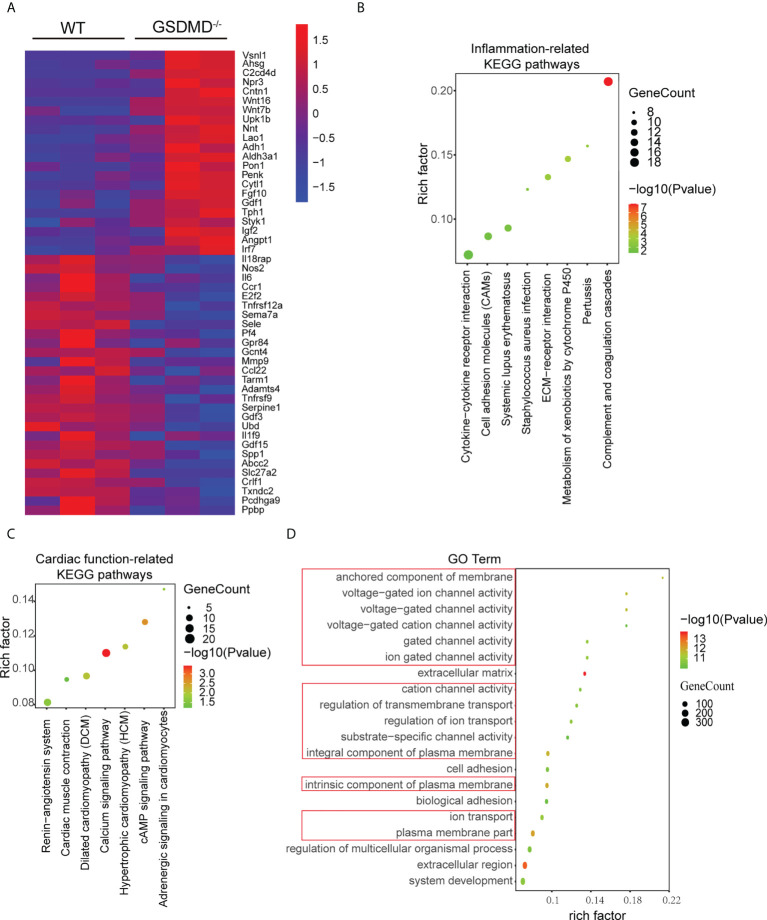
**(A)**, Heatmap showing significantly altered inflammation-related and apoptosis-related genes between WT mice and GSDMD^-/-^ mice on Day 3 after I/R. **(B, C)**, KEGG pathway analysis of inflammatory response-related genes **(B)** and cardiac function-related genes **(C)** in WT mice and GSDMD^-/-^ mice after surgery. **(D)** The results of GO analysis between WT mice and GSDMD^-/-^ mice after I/R.

To further clarify the mechanisms by which GSDMD deficiency protected against myocardial reperfusion injury, mRNA sequencing was performed on the infarct area in each group on Day 3 post I/R. Compared with those in the WT-I/R group, 1037 genes were upregulated significantly and 407 genes were downregulated in the GSDMD^-/–^I/R group. Heatmap analysis showed that the differentially expressed genes were mainly related to proinflammatory responses (such as Il-6 and Il1f9) and cell death (such as Tnfrsf9) (**Figure 7A**). Furthermore, Kyoto Encyclopaedia of Genes and Genomes (KEGG) pathway enrichment analysis showed that the most significantly altered pathways between the two groups were related to inflammatory responses (such as cytokine-cytokine receptor interactions and cell adhesion molecules) (**Figure 7B**), which indicated the vital role of GSDMD in the inflammation.

In addition, the KEGG results also showed that cardiac function-related pathways (such as the renin-angiotensin system and cardiac muscle contraction) (**Figure 7C**) were highly enriched. Among these pathways, enrichment of the calcium signaling pathway was the most significant and was closely related to the calcium ion concentration in cardiomyocyte. Calcium overload is one of the most important mechanisms in MI/RI. As a pore-forming protein, GSDMD disrupts the integrity of the membrane and alters cellular osmotic pressure, which affects calcium ion transport. Therefore, we performed Gene Ontology (GO) enrichment analysis to further examine the changes in molecular functions and biological processes. The GO analysis results (**Figure 7D**) showed that the top 20 GO terms were mainly related to plasma membrane composition, ion channel activity and transmembrane transport. These results suggested that GSDMD deficiency may regulate cardiac function by changing ion transport.

## Discussion

With the development and popularization of primary PCI, the mortality of AMI has decreased sharply. Restoring the occluded artery is the prerequisite for reducing infarct size. However, the reperfusion after an ischaemic episode causes another shock to living cardiomyocytes, leading to increased infarct sizes and even heart failure. Therefore, the therapeutic efficacy of AMI has been severely hampered. Since the concept of MI/RI was first described in 1960 (43), a large number of studies have been implemented to identify and explore protective interventions (44, 45). Despite over six decades of research, effective experimental interventions still cannot be translated into successful clinical applications. Given the complex underlying mechanisms and high clinical benefits, new insights and interventions are urgently needed.

Inspired by the importance of GSDMD in the release of IL-1β and clinical results, we studied the role of GSDMD in MI/RI. The results showed that GSDMD and GSDMD-NT upregulated after MI/RI, and GSDMD was mainly expressed in macrophage infiltrating in the infarct area. GSDMD deficiency considerably alleviated MI/RI and improved cardiac function by attenuating the release of inflammatory cytokines and the infiltration of neutrophils. The *in vitro* results further suggested the function of macrophages. Moreover, mRNA-sequencing analysis provided deeper perspectives on the potential mechanisms.

Many studies have demonstrated that both innate and adaptive immune responses are involved in MI/RI. DAMPs released by the injured myocardium trigger inflammatory responses. Dectin-1, a C-type lectin receptor, aggravates MI/RI by increasing the infiltration of inflammatory cells and the release of proinflammatory cytokines (37). Inhibiting TLR2, a Toll-like receptor, or knocking it out can all mitigate the inflammatory response, thus ameliorating MI/RI (46). However, we still do not know which specific ligands bind to these receptors and trigger inflammatory cascades. In addition, the downstream pathways that correlate with the release of various inflammatory cytokines have become the targets of interventions. IL-1β, the central cytokine in MI/RI, can recruit a large number of inflammatory cells, promote the synthesis of inflammatory cytokines and exacerbate myocardial damage. The administration of a recombinant IL-1 receptor antagonist reduced apoptosis and improved heart function after AMI (47). IL-18 (48) belongs to the IL-1 superfamily, and neutralizing IL-18 exerts cardioprotective effects after MI/RI (49, 50). Caspase-1 is responsible for IL-1β maturation and subsequent cell death, exerting a pernicious effect on MI/RI (51, 52). Due to the importance of these cytokines in MI/RI, any interventions that target them may be promising for treating this disease. In our study, knocking out GSDMD exhibited much lower serum levels of these two cytokines than WT mice after myocardial reperfusion injury, suggesting that GSDMD might be an effective intervention target. The latest researches (33, 34) showed that the loss of cardiomyocyte-specific GSDMD would reduce the infarct size and improve the heart function after MI/RI. Our study showed that the effect of pyroptosis on cardiomyocytes was limited and it suggested that there were other cells-mediated pyroptosis in MI/RI. Except for cardiomyocytes, there were endothelial, cardiac fibroblasts, smooth muscle cells and immune cells in the myocardium. The expression level of GSDMD in immune cells, such as monocytes and macrophages, were relatively higher than in other cells (53, 54). However, the research showed that infarct size after 45 min coronary occlusion was nearly final at the latest after 1 h reperfusion, way before many recruited inflammatory cells infiltrating (4, 55). So we focused on the tissue-resident immune cells and the research indicated that the resident macrophages were the most prominent population among cardiac leukocytes (56). Various researches had showed the important role of cardiac resident macrophages under physiology or pathology conditions (57, 58). IL-18, a critical cytokine in pyroptosis, was also observed in the resident myocardial macrophages (59). So we adopted macrophages in the *in vitro* experiments, and found that there were intense pyroptosis and incresed level of GSDMD cleavage in macrophages after H/R. Therefore, in view of the existence and function of resident macrophages in the myocardium, we supposed that the GSDMD on resident macrophages might be involved in MI/RI. These results enriched the mechanisms of GSDMD in MI/RI.

In addition to inflammatory cytokines, many proinflammatory mediators, such as SQSTM1 (60) and HMGB1 (61), can be released through GSDMD-mediated pyroptosis and aggravate inflammatory damage. According to the latest research, NLRP3 can be released *via* the pores formed by GSDMD-NT in hepatocytes and taken up by stellate cells, which leads to prolonged inflammation and intense fibrosis (62). Interestingly, before the rupture of the cell, GSDMD-NT can perforate the membranes of organelles, such as mitochondria and lysosomes, and cause the nonselective release of organellar proteins (63). GSDMD-NT oligomerizes on azurophilic granules and promotes the release of elastase into the cytosol, which in turn increases the cleavage of GSDMD (64). Besides, GSDMD-NT binds LC3^+^ autophagosomes and facilitates ATG7-dependent secretion of IL-1β (64). Furthermore, GSDMD can be activated by cleaved caspase-11 and perforate the nuclear membrane, causing the leakage of DNA and promoting the formation of neutrophil extracellular traps, which has been validated in many autoimmune diseases (65). GSDMD-NT can permeabilize mitochondria and activate caspase-3 by releasing cytochrome c (66). In addition, GSDMD-NT increases the production of ROS in mitochondria and activates inflammasomes (67). Except forming the classic membrane pore, GSDMD is involved in mediating the release of small extracellular vesicles containing IL-1β, which is a nonlytic effect (68). Therefore, more specific mechanisms of GSDMD in MI/RI need to be further explored.

However, there are still some issues requiring further in-depth investigations. First, despite functional data demonstrating the high expression of GSDMD in macrophages and the contribution of GSDMD to exacerbating MI/RI, we did not confirm the exact effect of GSDMD on macrophages during this process. In the future, we will perform bone marrow transplantation or macrophage-specific knockout to verify the cell-specific function of GSDMD. Second, we found that macrophages challenged with H/R or H_2_O_2_ exhibited obvious pyroptosis and upregulated expression of GSDMD-NT, and these findings need to be verified in GSDMD knockout cells. Third, although the level of GSDMD in the STEMI patients after PCI was higher than it in controls, the role of GSDMD in the clinical need to be investigated further.

## Conclusions

Our study showed that the loss of GSDMD would mitigate cardiac damage induced by myocardial ischaemia-reperfusion by inhibiting the release of IL-1β and IL-18 and the infiltration of neutrophils. These results suggested that GSDMD might be involved in I/R injury, which may provide a potential target for treatment.

## Data availability statement

The data presented in the study are deposited in the NCBI SRA repository, accession number PRJNA878781.

## Ethics statement

The studies involving human participants were reviewed and approved by the Institutional research Ethics Committee of Shanghai Minhang Hospital. The patients/participants provided their written informed consent to participate in this study. The animal study was reviewed and approved by the Animal Ethics Committee of the School of Pharmacy at Fudan University. Written informed consent was obtained from the individual(s) for the publication of any potentially identifiable images or data included in this article.

## Author contributions

XY and PZ designed and performed experiments, analyzed data, and wrote the paper; YZ performed experiments and analyzed data; JL analyzed and interpreted data; CX and ZW provided intellectual contribution; DJ and WH studied conceptualization, designed experiments, and obtained funding. All authors contributed to the article and approved the submitted version.

## Funding

This study was supported by grants from the National Natural Science Foundation of China (Grant No. 82073752), the Shanghai Science and Technology Fund (20JC1411000 and 20S11904700) and the Natural Science Foundation of Shanghai Municipality (19ZR1446000).

## Acknowledgments

We thank Dr. Feng Shao for sharing GSDMD^-/-^ mice. Besides, we thank Jiajun Fan for helping in designing the experiments.

## Conflict of interest

Author ZW was employed by TAU Cambridge Ltd.

The remaining authors declare that the research was conducted in the absence of any commercial or financial relationships that could be construed as a potential conflict of interest.

## Publisher’s note

All claims expressed in this article are solely those of the authors and do not necessarily represent those of their affiliated organizations, or those of the publisher, the editors and the reviewers. Any product that may be evaluated in this article, or claim that may be made by its manufacturer, is not guaranteed or endorsed by the publisher.
