# Supplementary material for: GSDMD contributes to myocardial reperfusion injury by regulating pyroptosis
Source: Front Immunol. 2022 Sep 23;13:893914. doi: 10.3389/fimmu.2022.893914 (PMC9546776; doi:10.3389/fimmu.2022.893914)
Supplement: Supplementary file 1 [file DataSheet_1.pdf]

Supplement Table 1: The baseline characteristics of the control group and AMI group.

	Control (n=23)	AMI (n=29)	*p* value
Age	63.04±2.52	64.66±2.50	0.656
Male, Sex	11 (47.83%)	26 (89.66%)	0.001
**CV risks**			
Hypertension	17 (73.91%)	16 (55.17%)	0.357
Diabetes	2 (8.70%)	11 (37.93%)	0.016
Dyslipidemia	8 (34.78%)	9 (31.03%)	0.775
Smoking	4 (17.39%)	16 (55.17%)	0.005
Smoking history	6 (26.09%)	20 (68.97%)	0.002
Drinking history	1 (4.35%)	6 (20.69%)	0.192
Systolic pressure (mmHg)	126.87±2.77	127.66±3.92	0.871
Diastolic pressure (mmHg)	78(70.00,90.00)	79(69.50,92.50)	0.637
Heart rate (beats/min)	78.48±2.23	88.38±4.19	0.043
**Lab. Examination**			
**Lipoproteins (mmol/L)**			
Triglyceride	1.46 (1.05, 2.05)	1.57 (1.09, 1.87)	0.985
Total cholesterol	4.08 (3.06, 4.69)	4.20 (3.70, 5.26)	0.381
LDL-C	2.65±0.16	3.12±0.21	0.102
HDL-C	1.02 (0.93, 1.33)	0.99 (0.87, 1.12)	0.269
Renal function			
BUN (mmol/L)	5.68±0.28	6.40±0.35	0.127
Creatinine (mmol/L)	69.00(63.00, 81.00)	80.00(66.00, 95.50)	0.107
Uric acid (mmol/L)	342.04±17.56	367.10±18.47	0.339
WBC (*10^9/L)	6.68±0.41	12.46±0.77	<0.001
Neutrophil (%)	60.10 (51.50, 67.90)	86.50 (80.35, 90.70)	<0.001
Stable HbA1c (%)	6.05 (5.58, 6.23)	6.10 (5.80, 7.30)	0.230
Serum glucose(mmol/L)	5.00 (4.50, 5.60)	6.50 (5.40, 8.95)	<0.001

CRP (mg/L)	2.99 (2.04, 4.83)	7.20 (6.95, 8.45)	<0.001
ESR (mm)	5.00 (2.00, 10.25)	10.30 (4.69, 25.55)	0.022
EF	0.63 (0.38, 0.65)	0.56 (0.49, 0.62)	0.319
**Discharge medication**			
Aspirin	15 (65.22%)	25 (86.21%)	0.074
Clopidogrel/ticagrelor	0 (0.00%)	25 (86.21%)	<0.001
ACEI/ARB	10 (43.48%)	7 (24.14%)	0.140
β-Blocker	10 (43.48%)	21 (72.41%)	0.035
Statin	17 (73.91%)	28 (96.55%)	0.049
Diuretics	3 (13.04%)	4 (13.79%)	1

The data are presented as the median (interquartile ranges) or n (%).

LDL, low-density lipoprotein; CV, cardiovascular; HDL, high-density lipoprotein; BUN, blood urea nitrogen; WBC, white blood cell; HbA1c, glycated haemoglobin; CRP, C reactive protein; ESR, erythrocyte sedimentation rate; ACEI, angiotensin-converting enzyme inhibitor; ARB, angiotensin receptor blocker

A

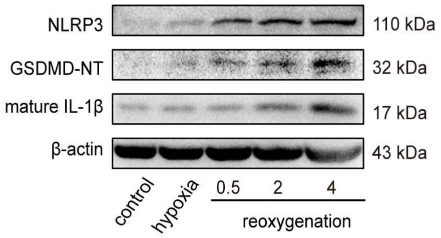

Figure S1

A, Immunoblot analysis of NLRP3, GSDMD-NT and mature IL-1β protein expression in H9c2 cells subjected to hypoxia for 6 hours and reoxygenation for different durations.

.

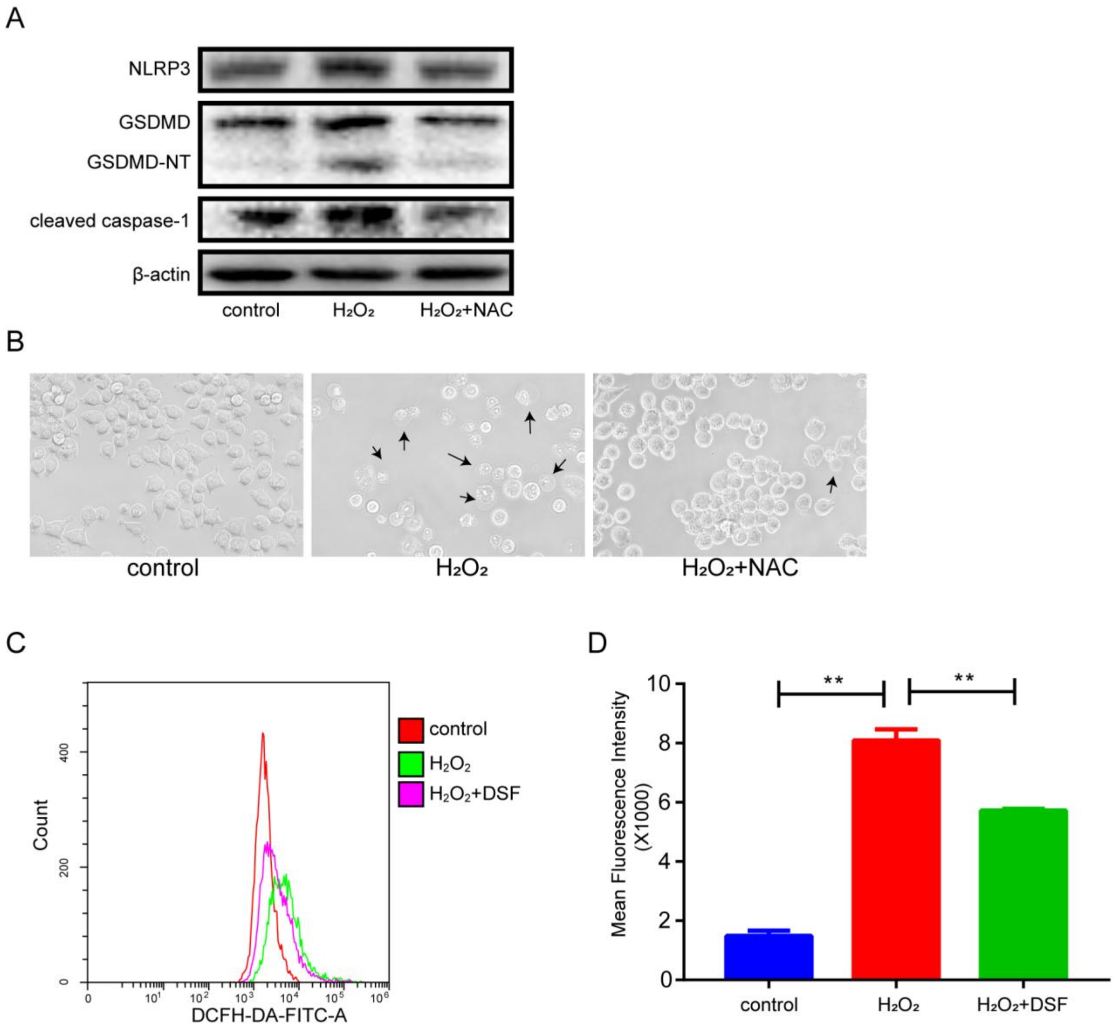

Figure S2

A , Representative Western blots (A) of NLRP3, GSDMD, GSDMD-NT and cleaved caspase-1 in Ana-1 cells treated with H_2O_2 or H_2O_2+NAC. B, Representative images of Ana-1 cells after H_2O_2 or H_2O_2+NAC treatment. C and D, Intracellular ROS levels were quantified using DCFH-DA for 30 min after the different treatments.
